# Deep-learning-based imaging-classification identified cingulate island sign in dementia with Lewy bodies

**DOI:** 10.1038/s41598-019-45415-5

**Published:** 2019-06-20

**Authors:** Tomomichi Iizuka, Makoto Fukasawa, Masashi Kameyama

**Affiliations:** 1 Center for Dementia, Fukujuji Hospital, Japan Anti-Tuberculosis Association, Kiyose, 204-8522 Japan; 2 Department of Nuclear Medicine, Fukujuji Hospital, Japan Anti-Tuberculosis Association, Kiyose, 204-8522 Japan; 3 Department of Diagnostic Radiology, Tokyo Metropolitan Geriatric Hospital, Tokyo, 173-0015 Japan; 4 Division of Nuclear Medicine, Department of Radiology, School of Medicine, Keio University, Tokyo, 160-8582 Japan

**Keywords:** Neurodegeneration, Alzheimer's disease, Alzheimer's disease, Diagnostic markers

## Abstract

The differentiation of dementia with Lewy bodies (DLB) from Alzheimer’s disease (AD) using brain perfusion single photon emission tomography is important but is challenging because these conditions exhibit typical features. The cingulate island sign (CIS) is the most recently identified specific feature of DLB for a differential diagnosis. The current study aimed to examine the usefulness of deep-learning-based imaging classification for the diagnoses of DLB and AD. Furthermore, we investigated whether CIS was emphasized by a deep convolutional neural network (CNN) during differentiation. Brain perfusion single photon emission tomography images from 80 patients, each with DLB and AD, and 80 individuals with normal cognition (NL) were used for training and 20 each for final testing. The CNN was trained on brain surface perfusion images. Gradient-weighted class activation mapping (Grad-CAM) was applied to the CNN to visualize the features that was emphasized by the trained CNN. The binary classifications between DLB and NL, DLB and AD, and AD and NL were 93.1%, 89.3%, and 92.4% accurate, respectively. The CIS ratios closely correlated with the output scores before softmax for DLB–AD discrimination (DLB/AD scores). The Grad-CAM highlighted CIS in the DLB discrimination. Visualization of learning process by guided Grad-CAM revealed that CIS became more focused by the CNN as the training progressed. The DLB/AD score was significantly associated with the three core features of DLB. Deep-learning-based imaging classification was useful for an objective and accurate differentiation of DLB from AD and for predicting clinical features of DLB. The CIS was identified as a specific feature during DLB classification. The visualization of specific features and learning processes could be critical in deep learning to discover new imaging features.

## Introduction

Neuroimaging has contributed to the classification of neurodegenerative dementias such as dementia with Lewy bodies (DLB) and Alzheimer’s disease (AD). Early diagnoses of DLB and AD are important from prognostic and therapeutic perspectives, and distinguishing them is clinically vital. Disease-specific features have been extracted from brain perfusion single photon emission tomography (SPECT) images to assist with differential diagnoses of DLB and AD. Brain surface perfusion images produced by three-dimensional stereotactic surface projection (3D-SSP)^1^ have been widely applied to statistical analyses that supported the diagnoses of DLB and AD. A perfusion decrease in the parietal association cortex (PAC) and a perfusion preservation in the primary motor and primary somatosensory cortex are typical in patients with DLB and AD^2,3^ and have interfered with distinguishing DLB from AD on perfusion SPECT images. An imaging feature for DLB discrimination is occipital hypoperfusion^4–7^. Another finding that can produce a difference between DLB and AD is perfusion in the posterior cingulate cortex (PCC). Hypoperfusion in the PCC is observed in the early stage of AD, whereas the PCC is relatively preserved in DLB. The phenomenon of sparing the PCC relative to the precuneus plus cuneus that is termed the cingulate island sign (CIS)^8^, has recently garnered attention because it reflects a concomitant AD pathology that affects the clinical symptoms of DLB^9,10^. We discovered CIS peaks at the stage of mild dementia and they disappear gradually as DLB progress^11^. Thus, the CIS can help differentiate DLB from AD especially at the early stage^8,12^ with some exceptions, including posterior cortical atrophy^13^.

Deep learning is a primary branch of artificial intelligence comprising a deep convolutional neural network (CNN) capable of automatic feature extraction from data, and recent advances in deep learning have remarkably improved the performance of image classification and detection^14,15^. Some algorithms based on deep learning have been proposed to recognize or differentiate AD and mild cognitive impairment (MCI)^16,17^. In contrast, the ability of a CNN to discriminate DLB has not been investigated in detail. Furthermore, a deep-learning-based SPECT interpretation system that can differentiate between DLB and AD has not been described. The most significant disadvantage of deep learning is that the imaging features used by the CNN for classification have remained unknown. However, gradient-weighted class activation mapping (Grad-CAM) can produce “visual explanations” from a CNN, thus allowing the visualization of areas focused by a CNN^18,19^.

The current study aims to investigate whether a trained CNN can identify the CIS, which is the most recently recognized imaging feature of DLB, while a deep two dimensional CNN (2D-CNN) objectively and automatically classifies brain surface perfusion images through the 3D-SSP of DLB, AD, and individuals with normal cognition (NL). Furthermore, the learning process was visualized during CNN training.

## Results

### Deep CNN could accurately classify brain surface perfusion images

**Figure 1:**
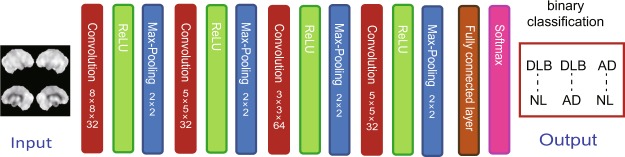
Architecture of deep convolutional neural network.

Tables 1 and 2 summarizes the demographic and cognitive findings of 80/20 persons, each with AD, DLB, and NL of the training/validation and final testing cohorts. The deep CNN was applied to images (*n* = 160) including the right-left flipped images from each group of 80 patients for binary classification (Fig. 1). The accuracy of the classification was calculated by the final testing cohorts. The binary differentiations between DLB and NL (DLB-NL), DLB and AD (DLB-AD), and AD and NL (AD-NL) were 93.07 ± 3.77%, 89.32 ± 4.59%, and 92.39 ± 4.42% accurate (mean ± standard deviation), respectively. The AUCs of the ROC for differentiating DLB–NL, DLB–AD, and AD–NL were 0.954, 0.935, and 0.943 accurate, respectively.

**Table 1:** Demographic features of study participants for training/validation.

	DLB	NL	AD
Participants (*n*)	80	80	80
Age (y)	77.7 ± 6.3	77.1 ± 6.8	78.0 ± 4.9
Sex (M/F)	44/36	40/40	36/44
MMSE score	22.8 ± 1.3*	29.5 ± 0.6	22.4 ± 1.9*
SPECT images (*n*)	160	160	160

Data are shown as numbers or means ± standard deviation. *p < 0.05: Tukey-Kramer test compared with NL (two-sided). DLB, dementia with Lewy bodies; NL, normal cognition; AD, Alzheimer’s disease; MMSE, mini-mental state examination; SPECT, single photon emission computed tomography.

**Table 2:** Demographic features of study participants for final testing.

	DLB	NL	AD
Participants (*n*)	20	20	20
Age (y)	77.9 ± 5.3	77.7 ± 5.0	77.8 ± 5.42
Sex (M/F)	11/9	9/11	9/11
MMSE score	22.5 ± 1.1	29.3 ± 0.7	22.3 ± 1.2
SPECT images (*n*)	40	40	40

Data are shown as numbers or means ± standard deviation. DLB, dementia with Lewy bodies; NL, normal cognition; AD, Alzheimer’s disease; MMSE, mini-mental state examination; SPECT, single photon emission computed tomography.

### Validation of epoch number and effect of sample number

**Figure 2:**
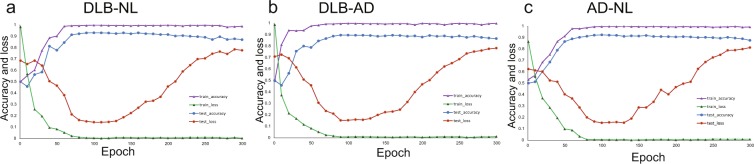
Learning curve. (**a**) DLB-NL, (**b**) DLB-AD, (**c**) AD-NL discriminations. Accuracy reaches plateau before 100 epochs. Test loss elevates gradually after 100 epochs, indicating overfitting.

One hundered epochs were confirmed to be suitable by the learning curve (Fig. 2).

**Figure 3:**
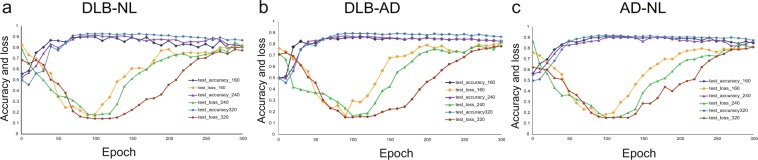
Effect of the sample number. (**a**) DLB-NL, (**b**) DLB-AD, (**c)** AD-NL discriminations. The accuracy did not differ greatly from the full set. However, smaller samples exhibited overfitting easily.

When the sample number was small, the accuracy did not differ greatly from the full set. However, smaller samples exhibited overfitting easily (Fig. 3).

### CIS ratios significantly correlated with DLB/AD and DLB/NL scores

**Figure 4:**
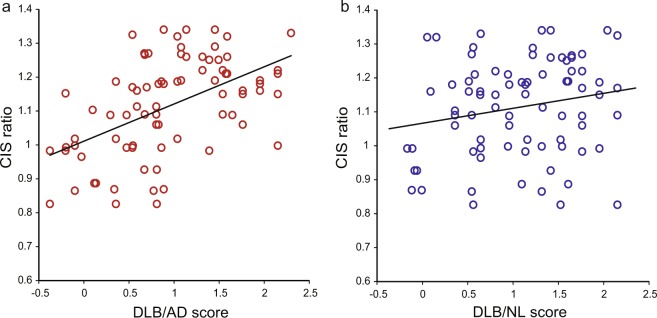
Association of CIS ratios with (**a**) DLB/AD and (**b**) DLB/NL scores. CIS ratio, DLB/AD score, and DLB/NL score in patients with DLB were 1.11 ± 0.14, 0.94 ± 0.67, and 1.08 ± 0.64, respectively (mean ± standard deviation). (**a**) CIS ratios correlated closely with DLB/AD scores (*r* = 0.511, *p* = 1.27 × 10^−6^). (**b**) CIS did not correlate with DLB/NL scores significantly (*r* = 0.195, *p* = 0.0835). CIS, cingulate island sign; DLB, dementia with Lewy bodies; AD, Alzheimer’s disease; NL, normal cognition.

Close significant correlations (Pearson’s product moment correlation) were found between the CIS ratios and scores for DLB/AD (*r* = 0.511, *p* = 1.27 × 10^−6^; Fig. 4a), whereas DLB/NL did not correlate with CIS significantly (*r* = 0.195, *p* = 0.0835; Fig. 4b) in patients with DLB. Thus, the CIS ratio contributed more to the differentiation of DLB–AD than of DLB–NL.

### Trained CNN identified CIS for DLB detection

**Figure 5:**
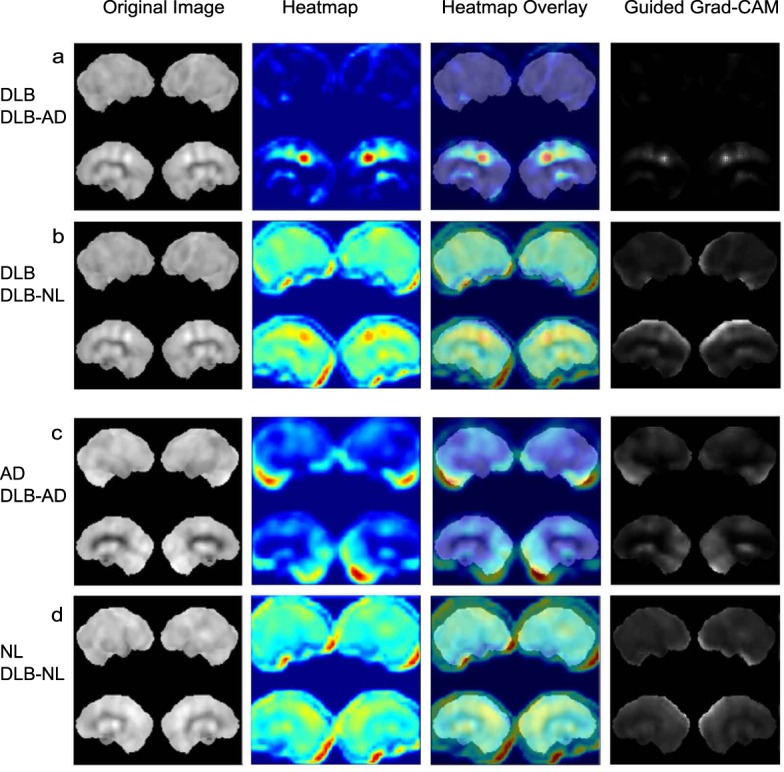
Visualization of features recognized by the trained CNN. Grad-CAM was applied to the CNN trained with 100 epochs, the produced heatmap, heatmap overlay, and guided Grad-CAM. Original and Grad-CAM images from one patient with DLB in the DLB–AD (**a**) and DLB–NL (**b**) discrimination, respectively. Original and Grad-CAM images from a patient with AD in the DLB–AD discrimination (**c**). Original and Grad-CAM images from an individual with NL in the DLB–NL discrimination (**d**). Original images of (**a**–**d**) were predicted correctly. CNN, convolutional neural network; DLB, dementia with Lewy bodies; AD, Alzheimer’s disease; NL, normal congition; Grad-CAM, gradient-weighted class activation mapping.

Grad-CAM was applied to the trained CNN to produce heatmaps and guided Grad-CAM images for DLB–AD and DLB–NL discrimination. The heatmap clearly highlighted CIS in DLB to discriminate DLB and AD (Fig. 5a). The guided Grad-CAM exhibited a limited range on the image that focused on CIS.

All the 80 DLB images are shown in the Supplementary Information. These images are arranged in the descending order of the DLB/AD score. CIS was highlighted in the first 61 DLB images. Among them, obviously highlighted CIS was found in the 48 images. Brain perfusion images with obvious occipital hypoperfusion without CIS were labeled correctly as DLB. Grad-CAM highlighted the cerebellum randomly. The last nine DLB images highlighted the occipital cortex without CIS and were mislabeled as AD.

CIS was highlighted less intensely in DLB–NL than in DLB–AD discrimination (Fig. 5b). The heatmap and guided Grad-CAM for AD highlighted the occipital lobe and cerebellum, but not the PCC (Fig. 5c). The heatmap and guided Grad-CAM for NL diffusely highlighted the occipital lobe, middle cingulate cortex, PCC, and cerebellum (Fig. 5d).

### Visualization of feature extraction in the learning process of CNN

**Figure 6:**
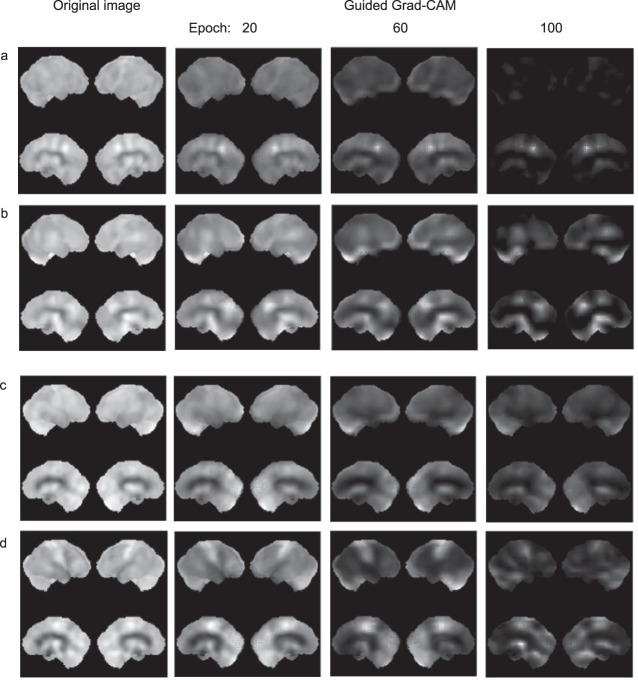
Alteration of guided Grad-CAM images in the learning process. Original and guided Grad-CAM images are from two patients, each with DLB and AD. Two patients, each with DLB (**a**) and (**b**), and AD (**c**) and (**d**). Training accuracies at 20, 60, and 100 epochs were 0.7682, 0.8922, and 0.9850, respectively. Validation accuracies at 20, 60, and 100 epochs were 0.6250, 0.7500, and 0.8750, respectively. Thus, 100 epochs were regarded as appropriate for training. The guided Grad-CAM images of both DLB and AD reduced with increasing number of epochs. Original images of (**a**–**d**) were predicted correctly. CIS, cingulate island sign; DLB, dementia with Lewy bodies; AD, Alzheimer’s disease; Grad-CAM, gradient-weighted class activation mapping.

Grad-CAM visualized the learning process to extract features that were useful for differentiation by displaying altered images (Fig. 6). In the CNN trained for DLB–AD discrimination with 20 epochs, guided Grad-CAM and original images remained similar, indicating that the CNN could not detect specific features. After training 60 epochs, the guided Grad-CAM images became narrower and the contrast became more obvious. After training with 100 epochs, the CNN focused more on CIS in DLB (Fig. 6a,b) and the occipital lobe, cerebellum, and sensorimotor areas in AD (Fig. 6c,d).

### DLB/AD score was associated with core features of DLB

The association between neuroimaging indices (*i.e*., CIS ratio, DLB/AD and DLB/NL score) and clinical symptoms (*i.e*., four core features and verbal memory) were analyzed. The DLB/AD score was significantly correlated with hallucination, Parkinsonism, and RBD, but not with fluctuation (Table 3). In contrast, the DLB/NL score was not correlated with any of them. The CIS ratio was correlated with hallucination and RBD. The DLB/AD score and CIS ratio were significantly correlated with verbal memory.

**Table 3:** Association between neuroimaging indices and clinical symptoms of DLB.

	Correlation coefficient		
	CIS ratio	DLB/AD score	DLB/NL score
Hallucination	0.307 (*p* = 0.0067^*^)	0.235 (*p* = 0.0231^*^)	0.203 (*p* = 0.0745)
Fluctuation	0.148 (*p* = 0.189)	0.117 (*p* = 0.303)	0.078 (*p* = 0.494)
Parkinsonism	0.104 (*p* = 0.367)	0.319 (*p* = 0.0033^*^)	0.212 (*p* = 0.0628)
RBD	0.450 (*p* = 2.8 × 10^−5*^)	0.268 (*p* = 0.0161^*^)	0.091 (*p* = 0.421)
Verbal memory	0.611 (*p* = 7.72 × 10^−10*^)	0.487(*p* = 4.69 × 10^−6*^)	0.201 (*p* = 0.0723)

Spearman rank correlation coefficients (two-tailed). *significant by Benjamini-Hochberg method with 0.05 of False Discovery Rate. CIS, cingulate island sign; DLB, dementia with Lewy bodies; AD, Alzheimer’s disease; NL, normal cognition; RBD, REM sleep behavioral disorder.

## Discussion

Our CNN identified the CIS as an imaging feature during DLB–AD discrimination. The CIS ratios correlated closely with the DLB/AD scores, indicating the possibility that the network assessed the CIS indirectly during the discrimination. Furthermore, heatmaps generated by the Grad-CAM highlighted the CIS in DLB. The guided Grad-CAM also focused on the CIS and became restricted to the CIS as the learning process progressed. The indirect evidence of the correlation coefficients may imply that a typical DLB possesses a higher CIS ratio. However, the trained CNN automatically and objectively identified the CIS as an important feature of DLB prediction, considering that the Grad-CAM could visualize the target of the CNN for the classification. The present findings defined the potential of deep learning to discover new features in image diagnosis.

The deep CNN could accurately classify brain surface perfusion images. The classification accuracies of DLB–NL, DLB–AD, and AD–NL were 93.1%, 89.3%, and 92.4%, respectively. Most previous studies using deep-learning-based classification aimed to diagnose AD and MCI but not DLB using 3D-CNN, and the CNN diagnosis of DLB using FDG PET or perfusion SPECT has never been reported. Suk *et al*.^17^ reported that the mean accuracies of MRI, FDG PET, and MRI + PET with 3D-CNN were 92.38%, 92.20%, and 95.35%, respectively. Liu *et al*.^16^ generated accuracies of 90.18% (MRI), 89.13% (PET), and 90.27% (MRI + PET). Our 2D-CNN with brain surface perfusion images extracted from whole brain perfusion SPECT data yielded comparable discriminative accuracy. The distribution on brain perfusion and glucose metabolism images was similar^20^. The bird’s-eye view brain surface perfusion images represent extracted features that are useful for discriminating neurodegenerative dementia. Furthermore, 3D-CNN requires more calculations to converge more parameters than 2D-CNN. Thus, 2D-CNN with brain surface perfusion images classified more efficiently than 3D-CNN with whole brain images. Our method, which can be operated in a standard computer, can potentially prevail in clinical settings.

The CIS was more involved in the discrimination of DLB–AD rather than of DLB–NL, considering the higher correlation coefficients of the CIS ratios and DLB/AD scores than the CIS ratios and DLB/NL scores. The Grad-CAM supported this notion by focusing on the CIS as an imaging feature of DLB in the DLB–AD and DLB–NL discrimination. Heatmap and guided Grad-CAM highlighted the CIS in the DLB-AD discrimination, while CIS was less highlighted in the DLB–NL discrimination. As DLB and AD exhibit common features such as rCBF decreases in the PAC, classification is typically more difficult for DLB–AD than DLB–NL. Most patients with DLB exhibit concomitant AD pathology^21^, which reportedly affects the CIS of patients with DLB. Specifically, the CIS is not obvious in DLB with abundant AD pathology. Similar to the CIS ratios, the DLB/AD scores in DLB reflects the degree of imaging features of AD that are presumably produced by concomitant AD pathology. Therefore, low CIS ratios and DLB/AD scores represent a high degree of concomitant AD pathology. Conversely, high CIS ratios and DLB/AD scores represent “pure” DLB. This explains why the CIS ratios exhibited a good correlation with the DLB/AD scores.

The Grad-CAM revealed that the CNN classified SPECT images in a manner unlike that of humans. Nuclear medicine physicians simultaneously evaluated these hypoperfused areas and preserved the regions to differentiate DLB from AD, and often considered the contrast of the preserved and decreased areas. In contrast, heatmaps generated by the Grad-CAM were placed only on regions with preserved rCBF in both AD and DLB in the appropriately trained CNN. Guided Grad-CAM images became narrower and restricted to more preserved regions as learning progressed. Consistent with these findings, the CNN focused only on the preserved regions to classify the brain surface perfusion images of both DLB and AD. Regardless of the classification method, the CNN still identified the CIS as an important imaging feature of DLB.

The DLB/AD score was correlated significantly with the scores of three core features, namely hallucination, Parkinsonism, and RBD. In contrast, DLB/NL score was not correlated with any of them. This finding suggested that the DLB/AD scores closely represented various symptoms of DLB. Similar to the DLB/AD score, the CIS ratio was also correlated with hallucination and RBD. As CIS has been reported to reflect AD pathology, a close correlation of the CIS ratio with DLB/AD score indicated that the DLB/AD score reflected comorbid AD pathology. Hallucination was frequently observed in DLB without AD pathology^22^. The manifestation of RBD was reportedly associated with less severe concomitant AD pathology^23^. Our finding was consistent with the previous reports demonstrating the association between core features and AD pathology. Furthermore, the DLB/AD score was correlated with verbal memory score, thus implying that memory impairment is prominent in patients with AD rather than those with DLB. Thus, the DLB/AD score was useful for both discriminating and predicting the clinical features of DLB.

Our deep learning system would be beneficial to health care finance. Dopamine transporter (DaT) imaging^24^ and [^123^I] MIBG cardiac sympathetic nerve scintigraphy^25^ are authentic in clinically discriminating DLB from AD, and the DLB guidelines treat DaT imaging and [^123^I] MIBG scintigraphy as indicative biomarkers^26^. However, to assess all amnestic patients using two more nuclear medicine examinations might be cost prohibitive. Brain perfusion SPECT is more commonly used to detect AD, especially when a diagnosis is uncertain. Consequently, our diagnostic system and perfusion SPECT could be initially applied to investigate DLB in patients with suspected AD before using DaT and cardiac sympathetic nerve imaging.

This study has several limitations. Each group comprised only 160 augmented images from 80 individuals because this study was performed at a single institution. However, our brain surface perfusion images were normalized by 3D-SSP and applied only to binary classification. Therefore, we considered that the accuracy was sufficient regardless of the limited number of patients. The accuracy of FDG PET might be better, but perfusion SPECT is more accessible, and it has been proven as a valid alternative in the absence of FDG PET^27^. Furthermore, images with [^123^I] IMP shows good contrast owing to its high first-pass extraction^11,28^. Recent CNN studies have attempted to enhance accuracy using various combinations of imaging modalities^16,17^. Although the ability of a 2D-CNN with brain surface perfusion images was comparable to previous findings with such combinations, combinations of perfusion SPECT with other imaging modalities should be considered in future studies to enhance accuracy.

## Conclusions

Deep-learning-based imaging classification was useful for an objective and accurate differentiation of DLB from AD, and for predicting the clinical features of DLB. The CIS was identified as a specific feature during DLB classification. The visualization of specific features and learning process could facilitate the discovery of new imaging features using deep learning.

## Methods

### Participants

Brain perfusion SPECT images of 80 persons, each with DLB, AD, and NL were included for diagnostic classification and CNN learning. Cognitive function was evaluated using the Clinical Dementia Rating and the Mini-Metal Status Examination (MMSE). Probable DLB and probable AD were diagnosed according to the McKeith criteria^26^ and the criteria of the National Institute for Neurological and Communicative Diseases Alzheimer’s Disease and Related Disorders Association^29^, respectively. Hallucination, fluctuation of cognition, Parkinsonism, and REM sleep behavioral disorder (RBD) were assessed by the Neuropsychiatric Inventory (NPI), Clinician Assessment of Fluctuation^30^, United Parkinson’s Disease Rating Scale-Motor Score (UPDRS-MS), and the Japanese version of the REM sleep behavior disorder screening questionnaire (RBDSQ-J)^31^, respectively. Verbal memory was evaluated using the sum of the five recall trials (1–5) of the Ray Auditory Verbal Learning Test (RAVLT).

Brain perfusion SPECT images of 20 persons each with DLB, AD, and NL were used for the final testing.

All procedures were approved by the Ethical Review Board of Fukujuji Hospital. We followed the clinical study guidelines of Fukujuji Hospital, which conformed to the Declaration of Helsinki (2013). We provided the healthy volunteers, patients, and their families with detailed information about the study, and all had provided written informed consent to participate in the study.

### Brain perfusion SPECT imaging

Persons resting with their eyes closed and ears unplugged were assessed by SPECT using Symbia Evo Excel, a gamma camera (Siemens Medical Solutions, Malvern, PA, USA), and fan beam collimators. Fifteen minutes after an intravenous infusion of [^123^I] IMP (167 MBq), SPECT images were acquired in a 128 × 128 matrix with a slice thickness of 1.95 mm (1 pixel) over a period of 30–40 min. The images were reconstructed by filtered back projection using a Butterworth filter; attenuation was corrected using the Chang method (attenuation coefficient = 0.1 cm^−1^), and scatter was corrected using a triple energy window. Brain surface perfusion images produced using 3D-SSP^1^ were augmented by flipping from left to right. The regional cerebral blood flow (rCBF) in the regions of interest (ROI) on the PCC, precuneus, and cuneus was measured as described^11^. The mean value in the bilateral PCC ROI was divided by the mean value in the bilateral precuneus plus the cuneus ROI to derive the CIS ratios from the [^123^I] IMP SPECT images. The CIS ratio is posterior cingulate/(precuneus + cuneus)^8^.

### Preparation for deep CNN

Figure 1 summarizes the architecture of our deep CNN. The network was built with Keras and TensorFlow (Google, Mountain View, CA, USA), a deep-learning framework. We selected simple structures as we found that deeper structures did not contribute to accuracy; we did not use transfer learning to visualize the learning process.

After the convolution operation, rectified linear unit (ReLU) and max-pooling operations proceeded on the output of convolution. The ReLU maintained positive input values whereas negative input values were changed to zeros. The max-pooling operation selected the maximum value and input this value into a smaller feature map. The input data were extracted from the brain perfusion SPECT images. The input image had a matrix of 200 × 200 pixels, *i.e.*, a composite of two lateral and two medial surface images. The input values of the voxels were rescaled within a range of 0 to 255; subsequently, the mean scalar value of each SPECT volume was subtracted. The images were passed through the first convolutional layer that produced 193 × 193 × 32 output images after the 8 × 8 × 32 convolutional filter. Thereafter, ReLU activation and the max-pooling of a 2 × 2 pool proceeded. The second convolutional layer with a 5 × 5 × 32 filter and 92 × 92 × 32 output was followed by the ReLU activation and max-pooling layers. The third convolutional layer with a 3 × 3 × 64 filter and 44 × 44 × 64 output was followed by the ReLU activation and max-pooling layers. The last convolutional layer with a 5 × 5 × 32 filter and 18 × 18 × 32 output was followed by the ReLU activation and max-pooling layers that produced a 9 × 9 × 32 output. Thereafter, a fully connected layer generated the output; subsequently, a softmax function was applied to discriminate the two labels.

The softmax produces two numerical values of which the sum becomes 1.0. The output values before softmax for the binary differentiation of DLB–NL, DLB–AD, and AD–NL are expressed as DLB/NL, DLB/AD, and AD/NL scores, respectively. We obtained the scores by applying an inverse sigmoid function to the output value. We employed binary discrimination to determine if the CNN recognizes the CIS differently in discriminating DLB–AD and DLB–NL. The network was trained to minimize cross entropy losses between the predicted and true diagnoses based on the images. We used the Adam optimizer and the proposed default settings (learning rate = 0.001, *β*_1_ = 0.9, *β*_2_ = 0.999, decay = 0.0) of the parameters^32^.

The CNN was trained for 100 epochs. The validity of the epoch number was verified by plotting the performance versus epochs. Furthermore, we plotted with reduced number of samples (0.5, 0.75 of original sample number (320)).

To visualize the decision made by the CNN, Grad-CAM was applied to the CNN. Grad-CAM uses the gradients of any target flowing into the final convolutional network to produce heatmaps that highlight important regions upon which the CNN focuses. A guided Grad-CAM was created by fusing the existing pixel-space gradient visualizations with Grad-CAM to achieve both high resolution and class discrimination. Furthermore, we used Grad-CAM to visualize the learning process of the CNN trained with perfusion images.

### Statistics

The diagnostic and predictive accuracy of the CNN was calculated from the independent final testing cohorts. An original image and its right-left flip image were in the same set of training or validation. Binary classification scores were evaluated using the receiver operating characteristic (ROC) curve analysis and area under the curve (AUC). Correlations between CIS ratios and DLB/AD or DLB/NL scores were assessed using Pearson’s product moment correlation coefficients. Correlations between clinical scores and CIS ratios, DLB/AD, or DLB/NL scores were assessed using Spearman rank correlation coefficients and the multiple comparison was assessed by the Benjamini-Hochberg method with 0.05 of False Discovery Rate. All statistical analyses were performed with EZR (Saitama Medical Center, Jichi Medical University, Saitama, Japan), which is a graphical user interface for R (The R Foundation for Statistical Computing, Vienna, Austria). More precisely, it is a modified version of the R commander designed to add statistical functions frequently used in biostatistics.

## Supplementary information

80 DLB images with Grad-CAM, arranged in the descending order of the DLB/AD score.
