# Supplementary material for: Deep-learning-based imaging-classification identified cingulate island sign in dementia with Lewy bodies
Source: Sci Rep. 2019 Jun 20;9:8944. doi: 10.1038/s41598-019-45415-5 (PMC6586613; doi:10.1038/s41598-019-45415-5)
Supplement: Supplementary file 1 — 80 DLB images with Grad-CAM, arranged in the descending order of the DLB/AD score. [file 41598_2019_45415_MOESM1_ESM.pdf]

Deep-learning-based imaging-classification identified cingulate island sign in dementia with Lewy bodies

Tomomichi Iizuka[1], **Makoto Fukasawa**[2], **and Masashi Kameyama**[3,4,*]

[1]Center for Dementia, Fukujuji Hospital, Japan Anti-Tuberculosis Association, Kiyose, 204-8522, Japan
[2]Department of Nuclear Medicine, Fukujuji Hospital, Japan Anti-Tuberculosis Association, Kiyose, 204-8522, Japan.
[3]Department of Diagnostic Radiology, Tokyo Metropolitan Geriatric Hospital, Tokyo, 173-0015, Japan
[4]Division of Nuclear Medicine, Department of Radiology, School of Medicine, Keio University, Tokyo 160-8582, Japan

[*]kame-tky@umin.ac.jp

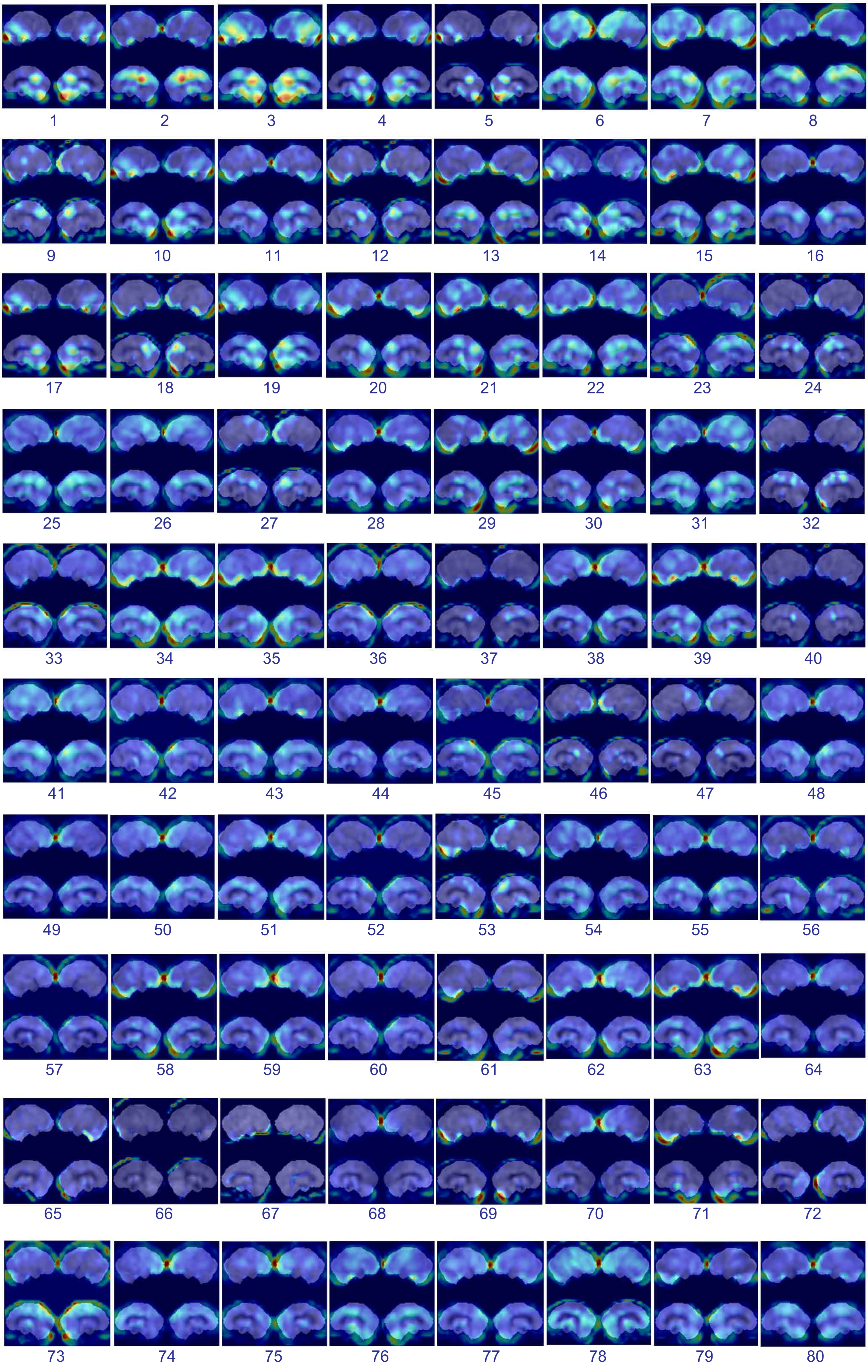
1
2
3
4
5
6
7
8
9
10
11
12
13
14
15
16
17
18
19
20
21
22
23
24
25
26
27
28
29
30
31
32
33
34
35
36
37
38
39
40
41
42
43
44
45
46
47
48
49
50
51
52
53
54
55
56
57
58
59
60
61
62
63
64
65
66
67
68
69
70
71
72
73
74
75
76
77
78
79
80
